# Fatty Acid Transport and Signaling: Mechanisms and Physiological Implications

**DOI:** 10.1146/annurev-physiol-032122-030352

**Published:** 2022-11-08

**Authors:** Dmitri Samovski, Miriam Jacome-Sosa, Nada A. Abumrad

**Affiliations:** 1 Center for Human Nutrition, Department of Medicine, Washington University School of Medicine, St. Louis, Missouri, USA; 2 Department of Cell Biology and Physiology, Washington University School of Medicine, St. Louis, Missouri, USA

**Keywords:** CD36, FFAR1, FFAR4, caveolin, fatty acid signaling, β-oxidation, tissue repair

## Abstract

Long-chain fatty acids (FAs) are components of plasma membranes and an efficient fuel source and also serve as metabolic regulators through FA signaling mediated by membrane FA receptors. Impaired tissue FA uptake has been linked to major complications of obesity, including insulin resistance, cardiovascular disease, and type 2 diabetes. Fatty acid interactions with a membrane receptor and the initiation of signaling can modify pathways related to nutrient uptake and processing, cell proliferation or differentiation, and secretion of bioactive factors. Here, we review the major membrane receptors involved in FA uptake and FA signaling. We focus on two types of membrane receptors for long-chain FAs: CD36 and the G protein–coupled FA receptors FFAR1 and FFAR4. We describe key signaling pathways and metabolic outcomes for CD36, FFAR1, and FFAR4 and highlight the parallels that provide insight into FA regulation of cell function.

## 1. INTRODUCTION

Long-chain fatty acids (FAs) are components of plasma membranes and complex cellular lipids such as triacylglycerols, phospholipids, and cholesterol esters. FAs are organic acids that are defined largely by their aliphatic side chain length and saturation. The side chains of animal FAs usually contain an even number of carbon atoms and are classified into short chains (2–6 carbon atoms), medium chains (8–12 carbon atoms), long chains (14–18 carbon atoms), and very long chains (20–26 carbon atoms). Most FAs in the circulation and tissues of mammals are long-chain and very-long-chain FAs. Among these are palmitic acid (C16:0), palmitoleic acid (C16:1), stearic acid (C:18:0), oleic acid (C18:1n-9), linoleic acid (C18:2n-6), arachidonic acid (20:4n-6), and docosahexaenoic acid (22:6n-3). The C16–C18 FAs are components of FA-derived signaling molecules (diacylglycerols and ceramides) and the long-chain, polyunsaturated FAs (e.g., arachidonic and docosahexaenoic acids) are precursors of major lipid signaling molecules (prostaglandins and leukotrienes).

FAs help maintain energy homeostasis by providing fuel through mitochondrial β-oxidation in times of energy need and by contributing to energy storage in times of plenty. The FAs are delivered to tissue cells through the circulation and cross the endothelial barrier to reach parenchymal myocytes, hepatocytes, adipocytes, and macrophages. In addition, FAs exert regulatory effects directly through interacting or associating with cellular proteins or through modifying cell membrane structure and function and as precursors to regulatory molecules with pleiotropic effects, such as eicosanoids and endocannabinoids. Impaired uptake and metabolism of FAs by tissues have been implicated in several conditions, including obesity-related insulin resistance and cardiovascular disease (1).

Over the past few decades, cellular FA uptake mechanisms have been the subject of investigation and intense debate focusing on whether transporters are needed to facilitate FA uptake as opposed to FAs diffusing passively across the membrane bilayer.

Circulating FA levels fluctuate throughout the day, and this is paralleled by rapid adjustments in FA uptake by tissues. For example, during fasting or prolonged exercise, as glucose availability diminishes, heart and muscle increase their reliance on FA uptake, which spares glucose for glucose-dependent tissues (2, 3). This suggests that tissue FA uptake is locally regulated to allow acute adjustments to changes in fuel supply. In agreement with this, early studies of FA uptake kinetics in intact cells (4–6) revealed uptake saturation with a half-maximal concentration (*K*_m_) below 10 nM of free FA and the presence of competitive inhibition between different FAs, consistent with a high-affinity, protein-facilitated transport process. The uptake *K*_m_, determined in isolated cells, was within concentrations of free albumin-dissociated FAs present in the circulation (7). Based on these findings, it was proposed that cellular FA uptake in vivo involves a saturable receptor-mediated process. However, at high free FA levels, which are presumed to occur at the endothelial cell surface during intravascular hydrolysis of triacylglycerol-rich lipoproteins, FA uptake could occur through loosening of intercellular spaces between cells (paracellular transport), as recently reviewed (8). FAs could also be ingested by cells (e.g., endothelial cells and macrophages) as components of unhydrolyzed lipoprotein particles (9).

Long-chain FAs in solution at physiological pH dissociate (ionize), donate a proton, and acquire a negative charge. The movement of ionized FA from one leaflet of the cell membrane to the other is slow, whereas nonionized FAs that have no charge can undergo rapid flip-flop. It has been proposed that ionized FAs partition into the outer membrane leaflet where they can get protonated, allowing flip-flop from the outer to inner leaflet of lipid bilayers. Studies that used protein-free lipid vesicles proposed that flip-flop is fast enough to mediate FA uptake into tissues. However, this interpretation has been challenged (as reviewed in 10). In a series of studies, Kleinfeld and colleagues (11–13) measured the flip-flop times of albumin-complexed FA by trapping fluorescent FA-binding proteins inside large vesicles or injecting them directly into cells. They concluded that membrane flip-flop times for long-chain FAs in larger vesicles and cardiomyocytes and adipocytes were too slow to accommodate cellular FA uptake rates, suggesting the need for protein facilitation. Although it is challenging to infer from in vitro studies whether proteins physiologically facilitate cellular FA uptake, protein facilitation of FA uptake is consistent with the need to manage FA tissue distribution in vivo. As FA availability changes with the metabolic state (e.g., fasted/fed, exercised, stressed), tissue uptake will not be efficient at low FA concentrations unless high-affinity mechanisms can direct FA to tissues with high FA requirements.

## 2. MEMBRANE FATTY ACID RECEPTORS

A compelling body of evidence supports protein facilitation of FA uptake, as recently reviewed (8). Several membrane or membrane-associated proteins [plasma membrane–associated fatty acid–binding protein (FABPpm), fatty acid transport proteins (FATPs), CD36] have been implicated in cellular FA transport and are discussed in this section. We focus on CD36, which has been the most widely studied, and its role in physiological FA uptake is supported by findings in cells, rodents, and humans. In Section 3, we discuss and compare FA-related signaling by CD36 and FA signaling by the well-studied G protein–coupled receptors (GPCRs) for long-chain FAs: FFAR1/GPR40 and FFAR4/GPR120. We discuss how signal transduction regulates the cellular fuel switch between glucose and FAs by influencing FA oxidation and insulin-stimulated glucose metabolism. Section 4 highlights a recently described role of increased FA uptake and FA oxidation in tissue maintenance, progenitor cell upkeep, and renewal after injury. Lastly, in Section 5 we discuss the pathological implications of low or dysfunctional FA sensing in humans, highlighted by genetic variants in the *CD36* and *FFAR* genes.

Transporters must reside on the plasma membrane to mediate the transmembrane movement of FAs and influence cellular lipid accumulation. Below, we discuss the role of FABPs, FATPs, CD36, and caveolin-1 (Cav-1) in facilitating FA uptake.

### 2.1. Plasma Membrane–Associated Fatty Acid–Binding Protein

FABPpm was identified in 1985 in jejunal microvilli and subsequently in several metabolic cells by oleate binding to plasma membranes and oleate-agarose affinity chromatography (14, 15). The protein was later found to be functionally identical to mitochondrial aspartate aminotransferase (16), and its targeting to the plasma membrane is thought to explain its role in FA uptake (17). In muscle, FABPpm is recruited to the membrane by insulin and muscle contraction to increase FA oxidation (18). In livers of obese mice (19), its levels correlate well with the V_max_ of FA transport. A model whereby CD36 acts in concert with FABPpm in mediating FA uptake has been proposed (reviewed in 20). However, a more recent study suggested the two proteins act independently. DNA injections of FABPpm and CD36 into rat muscles led to increased FA uptake in isolated giant sarcolemmal vesicles two weeks later. Effects on the efficiency of FA transport were additive when compared to overexpression of each protein alone, and CD36 immunoprecipitates did not contain FABPpm, arguing against a CD36–FABPpm interaction (21). Overall, recent studies of FABPpm have been limited, and how the protein might function in cellular FA handling at the membrane or mitochondria remains unclear.

### 2.2. Fatty Acid Transport Proteins (SLC27 Family)

The first member (FATP1) of the SLC27 protein family (FATP1–6) was identified in 1994 through a functional screening for FA import (22, 23). FATP1 expresses in adipose tissue, skeletal muscle, and heart; like other FATP proteins, it has acyl-CoA synthase activity that modifies and traps the FA inside the cell. FATPs are primarily intracellular proteins and likely to drive uptake by partitioning the FA metabolically. Tissue distribution and cellular localization of FATPs often determine their individual roles (24, 25). Endothelial cell FATPs were proposed to act downstream of vascular endothelial growth factor B (VEGF-B). The VEGFs maintain the microvasculature that controls nutrient exchange in tissues. Deletion of VEGF-B in mice was reported to reduce tissue FA uptake by lowering the expression of vascular FATP3 and FATP4 (26) and to reverse tissue lipid accumulation and glucose intolerance in diabetic mice (27). Other studies did not reproduce the effects of VEGF-B deletion on FA uptake or glucose tolerance. In contrast, VEGF-B overexpression in adipose tissue had beneficial metabolic effects through increasing adipose tissue vascularity and blood perfusion, which enhanced insulin delivery and action and the metabolic health of obese mice (28). Although the role of VEGF-B in endothelial FA uptake is now debated, the involvement of endothelial FATP in trapping FA through acyl modification was confirmed in a separate study. FATP4 was found to reside in endoplasmic reticulum (ER)/mitochondria microdomains and it promoted endothelial FA uptake through ATP-dependent acyl-CoA formation (29). Future work on expression and cellular localization of FATPs will help define the role of individual FATPs in tissue FA uptake.

### 2.3. CD36

**Figure 1:**
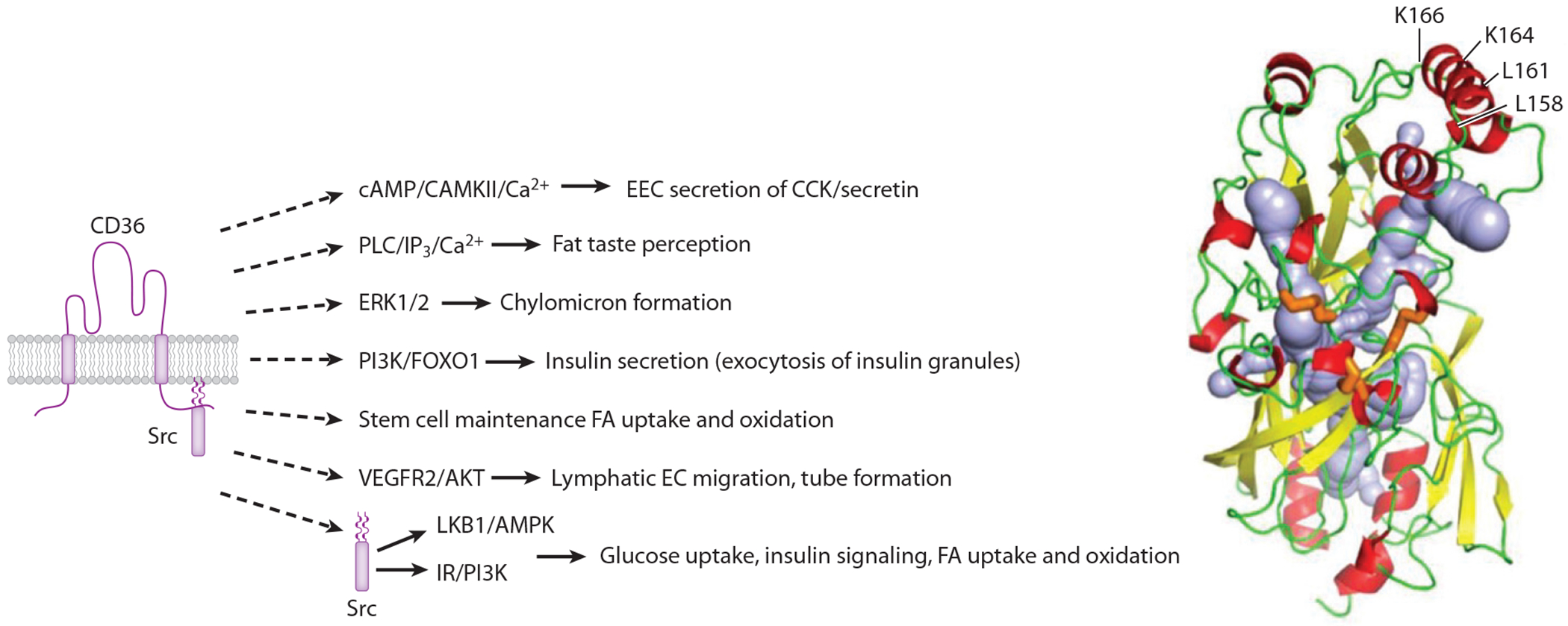
Metabolic pathways are controlled by long-chain FAs through CD36 signal transduction. FA binding to CD36 activates a number of signaling effectors that control FA metabolism: the cAMP/CAMKII/Ca^2+^ pathway in EECs modulates CCK/secretin secretion (93), the PLC/IP_3_/Ca^2+^ pathway is involved in gustatory detection of fat taste in taste buds (84, 97), ERK1/2 activation in enterocytes influences proteins active in chylomicron formation (45), PI3K/FOXO1 mediates insulin secretion in response to FAs in β-cells and pancreatic islets (90, 91), CD36-facilitated FA uptake and oxidation are required for gastric stem cell maintenance (86), VEGFR2/AKT is engaged in lymphatic endothelial cell migration and tube formation (109), and FA binding to CD36 modulates IR/PI3K and LKB1/AMPK signaling pathways, with effects on insulin signaling, FA and glucose uptake, and FA oxidation in skeletal myocytes (55, 82). (*Right*) Tertiary structure of CD36. α-Helices are shown in red, β-sheets in yellow, and the loops in green. Panel adapted from Reference 8. An intramolecular lipid transport tunnel was identified in CD36, and FAs were associated with the tunnel in crystal CD36. Abbreviations: AMPK, AMP kinase; CAMKII, Ca^2+^/calmodulin-dependent protein kinase II; cAMP, cyclic adenosine monophosphate; CCK, cholecystokinin; EC, endothelial cell; EEC, enteroendocrine cell; ERK1/2, extracellular signal-regulated kinases 1/2; FA, fatty acid; FOXO1, Forkhead box O1; IP_3_, inositol 1,4,5-trisphosphate; IR, insulin receptor; LKB1, liver kinase B1; PI3K, phosphatidylinositol 3-kinase; PLC, phospholipase C; VEGFR2, vascular endothelial growth factor receptor 2.

The role of CD36 (also known as SR-B2, GPIV, GPIIIb, PAS IV) in modulating FA uptake was shown in rat adipocytes using a reactive derivative of oleic acid, sulfo-*N*-succinimidyl oleate, which suppressed FA uptake and reacted with a 88-KDa membrane protein (30) later identified as CD36 (31, 32). CD36 is expressed on platelets, immune cells, adipocytes, myocytes, enterocytes, some enteroendocrine cells, and retinal and mammary epithelial cells. It is especially abundant on microvascular and lymphatic endothelial cells. CD36 recognizes FAs, lipoproteins, microbial lipids, and nonlipids (e.g., collagen, thrombospondin 1) and has pleiotropic cellular effects (reviewed in 33, 34). The crystal structure of CD36 family proteins (35) identified an intramolecular lipid transport tunnel. Long-chain FAs were shown to associate with the transport tunnel in crystal CD36 (36) and would reach the tunnel through a hydrophobic binding pocket on the CD36 surface (37) (Figure 1). CD36 has well-documented effects on FA uptake and metabolism, and these effects require its translocation to the plasma membrane, which can occur acutely in response to insulin or AMP kinase (AMPK), as recently reviewed (38).

CD36 resides in plasma membrane rafts and caveolae, with a hairpin topology spanning the bilayer twice and ending in two short cytoplasmic tails. Posttranslational modifications of CD36 affect its level of trafficking and therefore its function and include phosphorylation, glycosylation, palmitoylation, and ubiquitination. CD36 is reversibly S-palmitoylated (thioester bond of the palmitoyl with cysteine thiol) at the N- and C-terminal tails, which target it to the plasma membrane (39). Dynamic palmitoylation of CD36 by the palmitoyl acyl transferases DHHC4 and DHHC5 was shown to be required for its plasma membrane localization and for FA uptake activity (40). Restricting CD36 palmitoylation or depalmitoylation blocked its FA uptake activity. Glycosylation increases the apparent mass of CD36 from 53 kDa (not glycosylated) to 78–88 kDa and is essential for proper folding and trafficking to the plasma membrane (41). Stimulation of CD36 glycosylation by glucagon-like peptide-2 (GLP-2) stimulates intestinal lipid absorption and chylomicron production (42). Activation by glutamine of the hexosamine biosynthesis pathway has cardioprotective effects through enhancing O-linked N-acetylglucosamine (O-GlcNAc) protein modification, including that of CD36 (43). Glutamine treatment increases FA uptake/oxidation and membrane CD36 levels, but whether this is mediated by the enhanced O-GlcNAc modification of CD36 remains unclear. CD36 is ubiquitinated in response to FA exposure (44), intake of dietary fat (45), or exposure to platelet-derived exosomes (46), leading to a reduction of its levels. However, other types of ubiquitination stabilize CD36, as in the case of Parkin (47), or promote CD36 binding to insulin receptor substrate 1, thereby preventing its degradation (48). CD36 posttranslational modifications alter CD36 membrane localization or levels and could potentially be used for selective targeting of CD36 function.

Evidence for CD36 function in FA uptake was obtained by studies in mice and by validation of many findings in humans. In *Cd36*^−/−^ mice, FA uptake in heart, muscle, and adipose tissues is reduced (49), and this reduction is recapitulated by CD36 deletion specific to endothelial cells (50), consistent with the key role of the endothelium in tissue FA uptake. The *Cd36*^−/−^ mice also have delayed absorption of dietary fat and reduced intestinal lipid secretion into the lymph in addition to slow chylomicron clearance from the circulation (51). Human CD36 deficiency, observed in 9–10% of Africans and African Americans and 1–3% of Asians, results in defective uptake of FA by heart, muscle, and adipose tissues (52) and in higher blood FA levels (53). Relatively common CD36 variants that impact CD36 expression associate with altered chylomicron clearance, risk of metabolic syndrome, and type 2 diabetes (54, 55).

### 2.4. Caveolin-1

Cav-1 has been linked to FA uptake (reviewed in 56, 57). The caveolin family of proteins is abundant in adipocytes, myocytes, and endothelial and epithelial cells, but not in platelets or monocytes. Caveolin is the main structural component of caveolae and is involved in the formation of these flask-shaped invaginations 50–100 nm in diameter. Caveolae are specialized forms of rafts, with the membrane microdomains enriched in cholesterol and sphingolipids. The sphingolipids rich in long, mostly saturated FA chains form small free-floating domains, or rafts (58). Approximately 5% of the plasma membrane of mammalian cells consists of detergent-resistant membranes that can be biochemically separated by their insolubility in Triton X-100 at 4°C. Although sphingolipid-rich rafts are mainly in the outer leaflets, they are coupled with monounsaturated phospholipids on the cytoplasmic side. Cav-1 facilitates the assembly of caveolae through interaction with cholesterol (59) and FAs (60) and generally exerts inhibitory regulation of many caveolae-resident proteins through binding with its scaffold region (61, 62). Signaling proteins cluster in detergent-resistant membranes (rafts/caveolae), including membrane receptor kinases, insulin and epithelial growth factor receptors, protein kinases C and A, adenyl cyclase, mitogen-activated protein kinase intermediates (Ras, Raf, Sos, and Shc), heterotrimeric G proteins, and members of the Src family kinases. In the membrane, CD36 localizes to caveolae or rafts, and its function in FA uptake is dependent on this localization. CD36 function in FA uptake involves Cav-1 and the caveolae (63), and our unpublished data support the importance of the CD36 Cav-1 interaction in internalization of the FA with CD36.

Cav-1-deficient mice are resistant to high-fat-diet-induced obesity and have increased levels of triglycerides and FAs in the circulation (64). In addition to the membrane, Cav-1 localizes to lipid droplets and translocates to them during lipolysis (65, 66), suggesting its role in lipid trafficking. A more recent study (67) reported that endothelial cells lacking Cav-1 have enhanced lipolysis and impaired lipid droplet formation, which can be reversed by Cav-1 reconstitution. The decrease in lipid droplets was associated with a decrease in CD36 expression in blood vessels and isolated endothelial cells, suggesting that Cav-1 exerts its effect via CD36. Caveolae and Cav-1 may facilitate FA uptake by promoting CD36 localization and stabilization in the plasma membrane (68). Caveolae regulation of lipid metabolism has yet to be investigated in detail (69). Caveolin is abundant in microvascular endothelial cells as well as in adipocytes, two cell types involved in the management of lipid distribution. These cells engage in a metabolically regulated exchange of Cav1-containing extracellular vesicles (EVs) in vivo (70). Strong accumulating evidence indicates that EVs are a common channel for cross talk between cells or tissues through the exchange of cargo that can consist of lipids, signaling proteins, and regulatory microRNAs. Consequently, the EV exchange between the endothelial cells and adipocytes would allow transfer and exchange of plasma constituents and adipokines and will likely have pleiotropic metabolic effects in each recipient cell (71).

## 3. SIGNAL TRANSDUCTION BY MEMBRANE FATTY ACID RECEPTORS: FFARs AND CD36

Fatty acids, in addition to serving as an energy source, are important signaling molecules that control a wide range of cellular processes and physiological functions. It can be argued that signaling is a primary function for FAs, as it is critical to FA uptake at the plasma membrane. The downstream pathways recruited by FA membrane signaling control FA utilization and many of the metabolic actions of FAs. As described in the following sections, signaling by FAs is mediated by their membrane receptors, which function as signal transducers. We begin by summarizing signal transduction by the G protein–coupled FA receptors, also called FFARs, then discuss CD36 signaling and compare the effector pathways used by these two FA receptor types (Table 1).

**Figure 2:**
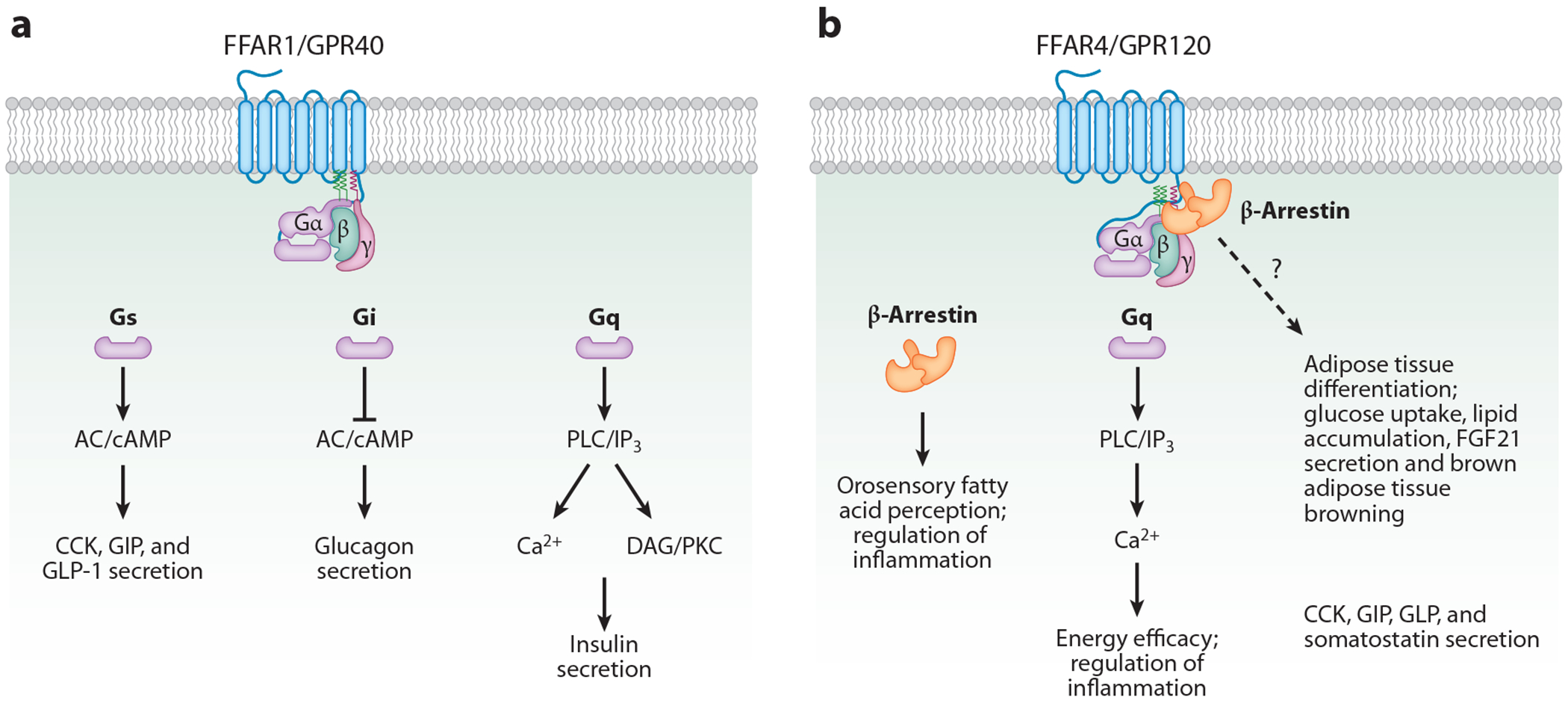
Metabolic pathways controlled by long-chain FAs through FFARs. By binding to (*a*) FFAR1/GPR40 and (*b*) FFAR4/GPR120, FAs mobilize several signaling effectors to control molecular pathways involved in FA metabolism: FFAR1 ligation by FAs activates the AC/cAMP pathway to regulate secretion of CCK, GIP, GLP-1, and glucagon; FAs regulate the PLC/IP_3_ pathway and modulate insulin secretion by binding to FFAR1; and FAs binding to FFAR4 play a role in fat taste perception, energy efficiency, inflammation, differentiation of adipose tissue, glucose uptake, lipid accumulation, brown adipose tissue browning, and the release of FGF21, CCK, GIP, and GLP, as well as somatostatin release. Abbreviations: AC, adenylate cyclase; cAMP, cyclic adenosine monophosphate; CCK, cholecystokinin; DAG, diacylglycerol; FA, fatty acid; FFAR1/GPR40, free fatty acid receptor 1/G protein–coupled receptor 40; FFAR4/GPR120, free fatty acid receptor 4/G protein–coupled receptor 120; FGF21, fibroblast growth factor 21; GIP, gastric inhibitory polypeptide; GLP-1, glucagon-like peptide-1; IP_3_, inositol 1,4,5-trisphosphate; PKC, protein kinase C; PLC, phospholipase C.

FFARs were discovered when FAs were found to bind GPCRs. Ligand binding to GPCRs causes the α subunits of receptor-coupled heterotrimeric G proteins to dissociate from the βγ subunits. The resulting effect of GPCR activation depends on which α subunit is engaged (Table 1 and Figure 2). Adenylate cyclase-mediated cAMP generation is one major effector of GPCR signaling through Gs-type Gα subunits and is inhibited by the Gi/o-type Gα subunits. The effector of the Gq/11α signaling is phospholipase C (PLC) which, when activated, hydrolyzes phosphatidylinositol 4,5-bisphosphate into diacylglycerol and inositol 1,4,5-trisphosphate (IP_3_), which in turn releases Ca^2+^ from the ER through binding IP3 receptors. GPCRs are inhibited by β-arrestins, which block the association of GPCRs to G proteins (reviewed in 72).

The FFAR family of receptors includes FFAR1 (GPR40), FFAR2 (GPR43), FFAR3 (GPR41), and FFAR4 (GPR120). Here, we focus on FFAR1 and FFAR4 that bind at micromolar concentrations to medium-chain (>C_6_) and long-chain (C_14_–C_22_) FAs, with FFAR4 biased toward long-chain FAs. Their physiological roles have been reviewed extensively (72–74) and are briefly highlighted here.

### 3.1. Free Fatty Acid Receptor 1

FFAR1 is expressed in pancreatic β-cells, immune cells, and intestinal enteroendocrine cells. It potentiates secretion of glucose-stimulated pancreatic insulin and gut incretins [glucagon-like peptide-1 (GLP-1), gastric inhibitory polypeptide (GIP), cholecystokinin (CCK)]. FFAR1 increases cellular cAMP through association with the Gs protein (75). FFAR1 is stimulated more by unsaturated FAs than by saturated FAs, with the polyunsaturated FA docosahexaenoic acid being most potent. Palmitic acid and palmitoleic acids are recognized ligands. Under physiological conditions, FFAR1 contributes approximately 50% of the FA-stimulated insulin secretion (76).

### 3.2. Free Fatty Acid Receptor 4

FFAR4 is expressed in taste bud cells, intestinal enteroendocrine cells, pancreatic islets, macrophages, and adipose tissue. FFAR4 couples to Gq, and its activation increases intracellular Ca^2+^. Unlike FFAR1, FFAR4 has not yet been linked to Gi or Gs proteins, but it has been implicated in taste perception and preference and in hormone secretion by the gut (GLP-1, CCK, GIP, peptide YY) and pancreas (insulin and glucagon). It has anti-inflammatory effects in macrophages where its activation by ω3 polyunsaturated FAs causes internalization of the β-arrestin-2 receptor (77) (Table 1 and Figure 2).

FFAR4 is reported to influence whole-body energy homeostasis (72, 73, 78). Unlike FFAR1, it is highly expressed in white adipose tissue and increases with high-fat feeding despite not being detected in preadipocytes (72). High-fat-fed FFAR4^−/−^ mice develop obesity, glucose intolerance, and fatty liver, and FFAR4 expression is increased in white adipose tissue of obese humans (79). A nonsynonymous mutation of FFAR4 (p.R270H), which impairs the receptor’s ability to mobilize intracellular Ca^2+^ in response to α-linoleic acid, was linked to increased obesity risk (79). FFAR4 is involved in thermogenesis, and its expression is increased by cold exposure, resulting in brown adipose tissue activation and white adipose tissue browning via fibroblast growth factor 21 (reviewed in 78).

The role of FFARs as candidate lipid sensors in regulating fat taste preference remains controversial. Initial reports of reduced FA preference in FFAR1- and FFAR4-deficient mice suggested that their upregulation might increase fat intake. However, only FFAR4 (not FFAR1) is confirmed to be expressed in taste buds of rodents and humans, but its role in fat preference remains unclear (72, 80). FFAR4 is expressed in the arcuate nucleus of the hypothalamus and the nucleus accumbens. Its acute activation by intracerebroventricular injection of an agonist in mice reduced food intake and suppressed rewarding effects of high-fat, high-sugar food (81).

### 3.3. Fatty Acid Translocase (FAT/CD36)

CD36 signaling was previously well documented in immune cells and more recently shown to be active in metabolic regulation (Table 1 and Figure 2). One of its notable actions is to regulate the cellular switch in fuel between glucose and FAs, which is impaired in *Cd36*^−*/*−^ mice. CD36 maintains low basal activity of AMPK and enhances basal insulin sensitivity through promoting Src phosphorylation of liver kinase B1 and that of the insulin receptor, respectively. FA binding to CD36 activates AMPK and suppresses insulin receptor signaling (55, 82), illustrating the role of FA sensing by CD36 in the regulation of energy homeostasis.

CD36 mediates taste perception of fats in mice (83) and humans (84). As reviewed elsewhere (80), in taste bud cells, FA binding of CD36 induces a rise in cytosolic Ca^2+^ , leading to the release of neurotransmitters, such as 5-hydroxytryptamine and noradrenaline, important in fat perception. FA/CD36 signaling involves PLC generation of the lipid mediators IP_3_ and diacylglycerol from phosphatidylinositol-4,5-bisphosphate and Ca^2+^ release from the ER through IP_3_-binding ER IP3 receptors. Subsequent activation of Ca^2+^ entry through plasma membrane store-operated Ca^2+^ channels increases cellular Ca^2+^ and ultimately neurotransmitter release to relay brain signals important for the cephalic phase of digestion and appetite regulation. CD36 expression is enriched in vagal neurons (85), and a lack of orosensory FA sensing in CD36 deficiency suppresses critical vagal input to the gut (86). The vagus-dependent (87, 88) early preabsorptive increase in blood insulin, pancreatic polypeptide, and leptin, measured 15 min after an oral high-fat meal, is markedly reduced in *Cd36*^−*/*−^ mice (86). Like FFAR4, CD36 is also expressed in the hypothalamus, where it influences neuronal FA-sensing and systemic metabolism. Its deletion from the ventromedial hypothalamus increased subcutaneous fat deposition and was associated with glucose intolerance (89).

In pancreatic β-cells, which express a specific CD36 transcript, the protein localizes to the plasma membrane and in insulin granules. Treatment with the CD36 inhibitor sulfo-*N*-succinimidyl-oleate blocked the acute effect of palmitic acid to stimulate insulin secretion. In addition, it blocked the effect of long-term palmitic acid exposure to inhibit glucose-dependent insulin secretion, indicating that CD36 mediates both stimulatory and inhibitory effects of palmitate on insulin release (90). However, in this study, the effect of chronic palmitate on CD36 levels in β-cells was not measured. It was later shown that inducible overexpression of CD36 in β-cells, while increasing FA uptake, blunts the integrated regulation of insulin secretion by glucose and FA, suggesting that high CD36 in islets has negative effects on exocytosis of insulin granules (91). In line with this, islets from obese donors with type 2 diabetes were found to have higher CD36 expression than obese nondiabetic donors, and this was associated with reductions in insulin secretion and β-cell exocytosis (92). The mechanism underlying the effects of high CD36 to impair insulin secretion was related to CD36 downregulation of β-cell insulin-signaling, which is predicted to inhibit docking and exocytosis of insulin granules. An antibody against CD36 increased expression of exocytosis-associated proteins and improved insulin secretion.

Together, the abovementioned findings suggest that, in islets, CD36 is on insulin granules and, like FFAR1, regulates the first phase of insulin secretion. However, its upregulation and dysfunction consequent to chronic FA exposure impair FA metabolism, notably FA oxidation, which is a well-recognized factor in the impairment of insulin secretion. In addition, the findings that CD36 resides on insulin granules and that its upregulation associates with the suppression of granule exocytosis suggest a role in granule trafficking and release, possibly involving signaling through Ca^2+^ or through cytoskeletal organization. Blocking islet CD36 in patients with type 2 diabetes might enhance insulin secretion, but a better understanding of its function in granule exocytosis will help design more selective approaches.

CD36 also regulates peptide secretion in a subset of enteroendocrine cells (93). It is found on secretin and CCK-positive enteroendocrine cells and is important for release of both hormones, which influence appetite and fat digestion. The response of plasma CCK and secretin levels to an oral lipid load was reduced by 50% in *CD36*^−*/*−^ mice. Together with FFAR1, CD36 accounts for CCK release induced by fat intake. In enteroendocrine cells (STC1 cells), CD36 interaction with FA (docosahexaenoic acid or linoleic acid) stimulates the release of CCK and secretin two- to threefold via increasing intracellular cAMP and Ca^2+^ levels (93).

**Figure 3:**
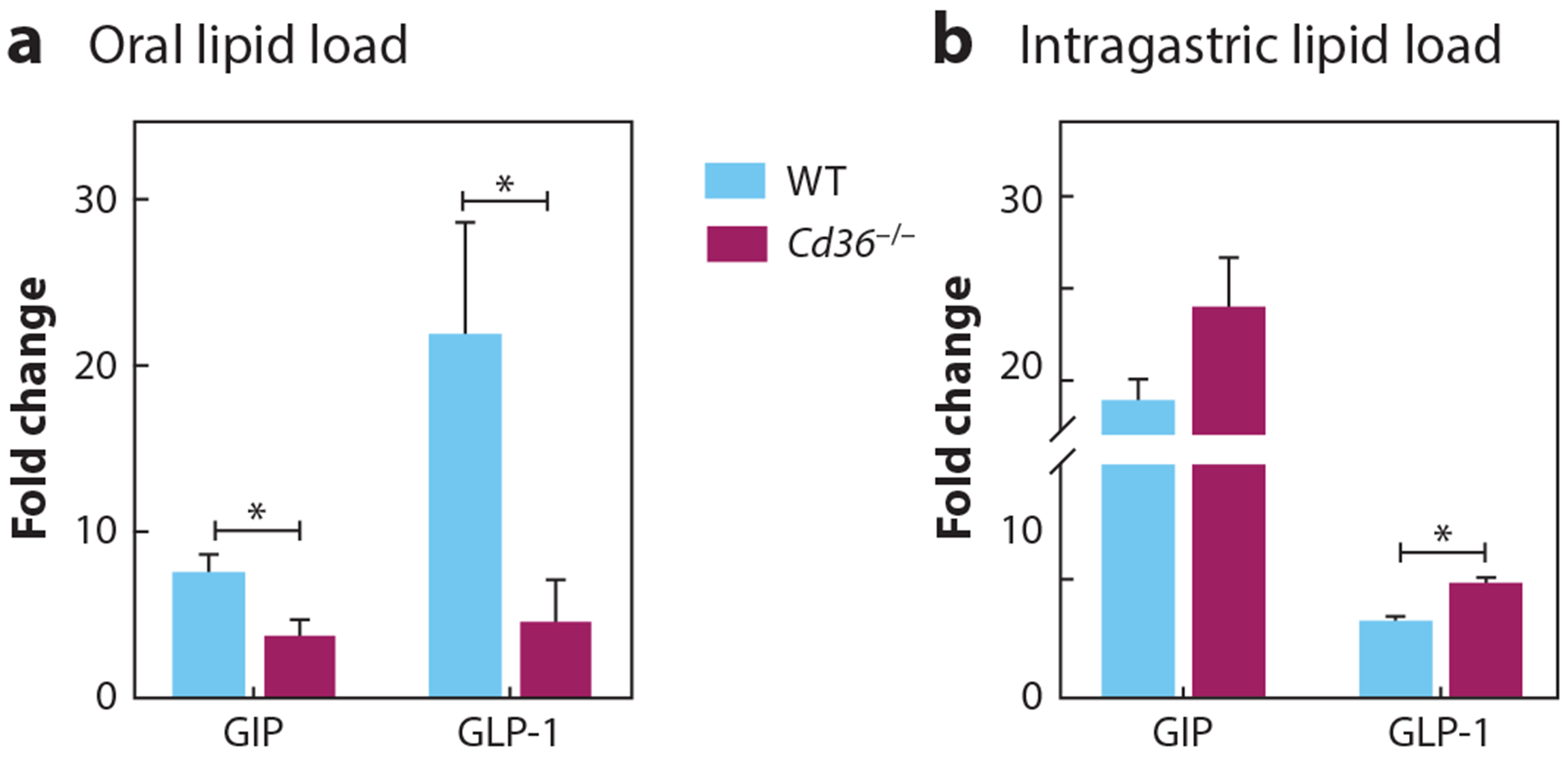
Opposite responses of gut incretin release after oral and intragastric lipid (olive oil) loads in *Cd36*^−*/*−^ mice. (*a*) Cephalic release of GIP and GLP-1 (relative to baseline) is suppressed in *Cd36*^−*/*−^ mice. The early cephalic response to food was determined at 15 min after oral exposure to a lipid load in overnight fasted WT and *Cd36*^−*/*−^ mice. (*b*) Intragastric olive oil or saline on incretin release measured in blood at 30 min. Plasma levels of GIP and GLP-1 in *Cd36*^−*/*−^ mice increased above those of WT mice. Data are expressed as fold change relative to saline control. *P < 0.05. Abbreviations: GIP, gastric inhibitory polypeptide; GLP-1, glucagon-like peptide-1; WT, wild-type.

As mentioned earlier, both FFAR1 and FFAR4 are potent stimulators of the release of GLP-1 and GIP by the gut (Table 1), but few studies have examined the role of CD36 in incretin release. The limited available findings suggest that it probably influences the release of GLP-1 and GIP in two ways. The preabsorptive cephalic response to food intake, important in priming the organism for efficient processing of a meal (94, 95), is impaired in *Cd36*^−*/*−^ mice (83, 86) and in partially CD36-deficient humans (96). This is in line with the established role of CD36 in orosensory FA perception in mice (83) and humans (84). The early preabsorptive release of GLP-1 and GIP is blunted in *Cd36*^−*/*−^ mice (Figure 3a) and in partially CD36-deficient humans (96). In contrast, when the FA is given intragastrically, thereby bypassing the orosensory/vagal system, CD36 deletion associates with the enhanced release of GIP and possibly GLP-1, suggesting that in vivo at the level of enteroendocrine cells, CD36 inhibits FA-induced release of the incretins (Figure 3b). Together, these data suggest that CD36 plays a role in the early preabsorptive release of incretins that primes the organism for fat absorption and tissue uptake. However, once digestion proceeds in the gut, CD36 on enteroendocrine cells might act as a decoy receptor recruiting FAs away from FFARs. Deleting CD36 makes FAs more available to the FFARs, enhancing their effect on incretin secretion. This is supported by the observation that CD36 in taste bud cells functions at much lower FA concentrations than does FFAR4 to induce Ca^2+^ signaling (97).

In contrast to CD36, the FFAR1 and FFAR4 receptors do not express on enterocytes, so their effects on fat absorption would be exerted through regulating secretions by enteroendocrine cells. CD36 is abundant on enterocytes, and its signal transduction regulates processing of a high-fat meal through promoting prechylomicron transport vesicle budding and exit from the ER to the Golgi (98) and through the upregulation of key proteins for chylomicron formation (45). Luminal lipids, most likely FAs, induce ubiquitination and degradation of CD36, as the protein disappears from the luminal side of villi early during the postprandial period (45). This regulation is impaired by high-fat feeding and the associated hyperinsulinemia, which blocks CD36 ubiquitination and results in postprandial hypertriglyceridemia (99). CD36 deletion delays lipid absorption with more fat reaching the distal gut and increases the generation of smaller remnant-like chylomicrons that are slowly cleared from the circulation. The deletion also suppresses lipid secretion into the lymph and causes more lipid to rapidly appear in the circulation, bypassing lymphatic transport (51, 100). Recent findings discussed below argue that these effects reflect the lack of CD36 signaling that maintains endothelial and epithelial barriers.

Overall, the similarities in the signaling effects of CD36, FFAR1, and FFAR4 suggest that a better understanding of their potential interaction in regulating long-chain FA actions is needed. In contrast to CD36, FFAR4 does not enhance FA uptake (101), but there is evidence that FFAR4 internalizes in response to FA stimulation. It is not known whether this process associates with simultaneous FA internalization, as in the case of CD36 (102). In pancreatic β-cells, the FFARs might not modulate metabolism of the FA, but they could play a role in mitigating the negative effects of abnormal FA metabolism and consequently the impairment of insulin secretion due to dysfunctional CD36. The potential of the synergistic effects of FFAR1 and CD36 in islets has not been tested. Functional synergy between CD36 and FFARs could occur based on the differences in FA specificity, FA affinity, and the expression profiles of the receptors.

## 4. CD36 SIGNALING AND FATTY ACID OXIDATION IN TISSUE MAINTENANCE AND REPAIR

Recent work highlighted an important role of CD36 signaling and the upregulation of FA oxidation for optimal tissue maintenance and function, which help reduce the risk of pathology and disease. Disruption of the gastrointestinal barrier notably by high-fat diets, infection, and damaging agents results in a leaky gut, which has been linked to the etiology of multiple gastrointestinal diseases and the systemic metabolic dysfunction caused by overnutrition (103, 104). A number of CD36 functions unrelated to FA metabolism, such as mounting a full immune response to enteric pathogens (105) and resolving inflammation through facilitating neutrophil clearance (106, 107), could help promote gut homeostasis. Recently, CD36 expression was shown to be required for maintaining integrity of the intestinal and endothelial barriers (108). CD36 deletion leads to extracellular matrix accumulation, compromises epithelial intercellular junction complexes, and causes neutrophil infiltration and inflammation of the small intestine. This phenotype of *Cd36*^−*/*−^ mice is mostly reproduced with endothelial cell CD36 deletion, suggesting that the CD36-deficient endothelium might promote neutrophil infiltration with the consequent loss of epithelial barrier integrity. A recent study (109) documented the role of CD36 signaling and FA oxidation in the regulation of the lymphatic endothelial barrier. CD36 expresses in lymphatic capillaries (lacteals) and is especially abundant in collecting lymphatic vessels. Its deficiency in lymphatic endothelial cells impaired intercellular VE-cadherin junctions that appeared fragmented and reduced in density, causing lymph leakage, visceral obesity, and systemic glucose intolerance. Silencing of CD36 in cultured human dermal lymphatic endothelial cells showed that CD36 is required for the metabolic switch toward increased FA oxidation through its modulation of VEGF-C signaling to VEGFR2/AKT. The increase in FA oxidation is critical for fueling cell migration and tube formation during lymphangiogenesis (110, 111) and for regulating VE-cadherin junction morphology (112) and stabilization (113). These findings support a critical role of CD36-mediated FA oxidation in regulation of the endothelial barrier. In this context, systemic carnitine palmitoyltransferase 1α (CPT1α) inhibition with etomoxir increased blood vessel leakiness and altered Ca^2+^ homeostasis (114). These findings might have relevance to the mechanisms underlying the endothelial dysfunction observed in humans with partial CD36 deficiency, where reduced flow-mediated dilation that was insensitive to treatment with the nitric oxide-cyclic guanosine monophosphate potentiator sildenafil was observed (53).

One of the most interesting new findings in tissue homeostasis is the role of FA oxidation in regulating progenitor cell function and tissue maintenance. For example, the peroxisome proliferator–activated receptor delta (PPARδ, also known as PPARβ)-mediated induction ofa gene network for FA oxidation is critical for stem cell function across multiple cell types (115). FA oxidation promotes stem cell maintenance in hematopoietic and neural stem cells (116, 117), and its inhibition impairs its ability for optimal differentiation and long-term renewal of proliferative intestinal stem cells (118). In endothelial cells, which utilize FA-derived carbon for synthesis of deoxyribonucleotides and DNA during cell replication, CPT1α silencing impairs endothelial cell proliferation (111). An intriguing new research area relates to the ability of EVs to transfer mitochondria between cells, which might play a role in tissue maintenance. In diet-induced obesity in mice, the energetically stressed adipocytes release into the circulation EVs that contain respiration-competent although oxidatively damaged mitochondrial particles. Uptake of these EVs by cardiomyocytes results in a compensatory increase of antioxidant signaling that limits heart injury from ischemia reperfusion (119).

Our recent data highlighted the abovementioned pathway in the stomach, which was found to be functionally dependent on the robust uptake of circulating FAs. CD36 expression is high in the stomach corpus and especially abundant in endothelial and parietal cells. In parietal cells, CD36 localizes to the basolateral site adjacent to capillary endothelial cells and is excluded from the luminal side and the ingested food, which contrasts to the luminal-facing CD36 on enterocytes of the small intestine. Using the acute injury model of high-dose tamoxifen (120), which causes gastric parietal cell–targeted death followed by rapid de novo parietal cell renewal, we found that endothelial FA delivery was important for stem cell differentiation and gastric epithelial renewal after injury (86). *Cd36*^−*/*−^ mice exhibited reduced parietal cell recovery despite active progenitor cell proliferation. This phenotype was related to reduced endothelial cell FA delivery to the corpus, which caused extensive remodeling of tissue lipids, diminished tissue respiration, and inefficient mitochondrial FA oxidation. Thus, deficiency of CD36-mediated FA uptake and oxidation in the corpus results in parietal cell progenitor cells incapable of full differentiation. Unlike *Cd36* deletion in endothelial cells, its deletion in parietal cells did not reduce corpus FA uptake or impair parietal cell differentiation, indicating that impaired mucosal renewal was not related to dysfunction of mature parietal cells. CD36 is a PPAR target, and CD36-mediated FA uptake activates PPARδ (121); thus, CD36 is likely to contribute to the effect of PPARδ on stem cell differentiation. CD36-mediated FA uptake by hematopoietic stem cells during acute infection was shown to favor a metabolic switch from glycolysis to FA oxidation, which enhances stem cell replication and organismal survival during infection (122).

## 5. PATHOLOGICAL IMPLICATIONS

The alterations in FA sensing and lipid metabolism observed in *Cd36*^−*/*−^ deficient mice are recapitulated in humans with partial or total CD36 deficiency. CD36 variants, common across ethnicities (39–45%), have been linked to elevated plasma lipids and to an increased risk of metabolic syndrome (54, 123–125) and stroke (126). Total CD36 deficiency in people (127) and CD36 single nucleotide polymorphisms (SNPs) or DNA methylation sites that reduce protein expression (54, 128) are associated with higher levels of chylomicron remnants and low density lipoprotein particles in blood, two risk factors of heart disease. Subjects carrying common CD36 SNPs display a greater oral lipid detection threshold (reduced fat taste perception) (84), decreased early cephalic responses to a meal (96), increased acceptance of added fats (129), and higher caloric intake attributed to more fat consumption (130, 131). Furthermore, exploration of RNA sequencing linked to whole genome data in humans (from the Vanderbilt BioVu and UK Biobank databases) identified associations between genetically determined low tissue CD36 expression and the incidence of type 2 diabetes and its associated complications (55) and the incidence of gastrointestinal diseases and risk of death from gastrointestinal hemorrhage (86).

Associations between variants in FFARs and metabolic disease have been less consistent and less well studied. FFAR4 SNPs were shown to modulate the effect of ω3 FAs on insulin-related traits, with fish oil supplementation improving these traits in noncarriers as compared to carriers of the minor alleles (132). A genetic variant in the *FFAR4* gene (p.R270H), which impairs receptor signaling, was associated with obesity in one study but not another (79, 133). A loss-of-function mutation of the *FFAR1* gene was associated with lower stimulated insulin secretion in humans (134), but so far no genetic variants have been linked to increased diabetes risk.

Collectively, these findings support the role of low or dysfunctional CD36 in the etiology of abnormal lipid uptake and impaired adaptation to energy transitions or cellular stress, which increases risk of metabolic abnormalities and chronic disease. The relationship of FFAR1 and FFAR4 variants to obesity-associated metabolic disease is intriguing and warrants further investigation.

## 6. CONCLUSION AND FUTURE DIRECTIONS

The metabolic implications of low or dysfunctional CD36 have been highlighted by genetic association studies, but little is known about CD36’s role in human tissue metabolism and repair. It is unclear what tissues primarily drive the metabolic abnormalities associated with low or dysfunctional CD36. Although disease-related common CD36 SNPs associate with low tissue CD36 expression, several studies have shown that high plasma CD36 levels correlate with greater abdominal adiposity (135) and liver fat (136, 137). Plasma CD36 likely reflects the presence of the protein in micro- or nanoparticles shed by cells, including platelets, that might be indicative of dysfunction and an abnormal metabolic status (138). Chronic upregulation of CD36, as well as low or complete absence of CD36 expression, would exert negative effects, as both conditions reduce the normal adaptive and homeostatic response that the protein mediates. Little is known about regulation of the CD36 promoter in humans that might exert tissue-specific influence. Alterations in intracellular trafficking and posttranslational processing of CD36 might also contribute to differential tissue-specific CD36 functions.

Recent data from our group documented that the endothelium is a major site of regulation for tissue FA uptake, and this applies to heart, skeletal muscle, and adipose tissue (50). Our unpublished data on the mechanism of endothelial FA handling suggest that dysfunction of FA uptake at the level of the endothelium could help drive metabolic abnormalities in other organs, a possibility that would be important to investigate. Additional investigation of the interactions between CD36 and FFAR1 and FFAR4 in determining various physiological outcomes related to insulin secretion or to intestinal lipid processing is warranted and could enhance our knowledge of the etiology of obesity-associated metabolic diseases.

## Figures and Tables

**Table 1:** FFAR1, FFAR4, and CD36: distribution, effectors, and metabolic outcomes

Signaling effectors	Expression	Metabolic functions	References
**FFAR1/GPR40**			
Gq	Pancreatic β-cells	FA enhancement of glucose-stimulated insulin secretion	139, 140
Gi	Pancreatic α-cells	Stimulation of glucagon secretion	139, 141, 142
Gs	EECs: I, K, and L cells	Stimulation of CCK, GIP, and GLP-1 secretion	143
**FFAR4/GPR120**			
Gq	Hypothalamus	Energy efficacy, regulation of inflammation	144
β-Arrestin	Taste buds	Orosensory FA perception	145
Not established	White adipocytes	Adipocyte differentiation, glucose uptake, lipid accumulation	77, 79
Not established	Brown adipocytes	BAT activation, FGF21 secretion	146
Not established	EECs: I, K cells	CCK, GIP, and GLP-1 secretion	147–149
Not established	Pancreatic δ-cells	Somatostatin secretion	150
β-Arrestin	Macrophages	Anti-inflammatory effect of DHA	77
**CD36**			
Src/LKB1/AMPK	Myotubes, muscle	FA uptake and oxidation	82
Src/IRβ/IR	Myotubes, muscle	Glucose uptake, insulin signaling	55
Not established	Pancreatic β-cells	FA regulation of insulin secretion	90
cAMP and Ca^2+^	EECs: I and S cells	CCK and secretin secretion	93
ERK1/2	Enterocytes	Chylomicron formation	45
Src/PLC/Ca^2+^	Tongue, taste buds	Neurotransmitter release, fat taste perception	97
Not established	Vagal neurons	Cephalic phase	85
VEGFR2/AKT	LECs	LEC FA oxidation, LEC migration, tube formation	109
PPARδ	Stem cells	Progenitor differentiation in tissue repair, stem cell replication for survival during infection	86, 122
Ca^2+^	Hypothalamus	More subcutaneous fat glucose intolerance	89

Abbreviations: BAT, brown adipose tissue; cAMP, cyclic adenosine monophosphate; CCK, cholecystokinin; DHA, docosahexaenoic acid; EEC, enteroendocrine cell; ERK1/2, extracellular signal-regulated kinases 1/2; FA, fatty acid; FFAR1, free fatty acid receptor 1; FFAR4, free fatty acid receptor 4; FGF21, fibroblast growth factor 21; GIP, gastric inhibitory polypeptide; GLP-1, glucagon-like peptide-1; GPR, G protein–coupled FA receptor; IRβ, insulin receptor beta; LEC, lymphatic endothelial cell; LKB1, liver kinase B1; PLC, phospholipase C; PPARδ, peroxisome proliferator–activated receptor delta; VEGFR2, vascular endothelial growth factor receptor 2.

## SUMMARY POINTS

1. Long-chain fatty acids (FAs) are membrane components, serve to provide fuel and bioactive molecules, and influence basic cell functions such as proliferation, differentiation, migration, and secretion by signaling through FA membrane receptors.
2. Among membrane FA transporters, strong evidence supports the role of CD36 and caveolin-1. Membrane transport is coupled to FA-acyl synthases, e.g., the fatty acid transport proteins (FATPs) that initiate cellular FA processing.
3. CD36 translocation to the plasma membrane and its internalization with FAs are regulated by posttranslational modification, particularly dynamic palmitoylation.
4. CD36/FA signaling regulates fat handling: orosensory FA perception in taste cells, cephalic secretions that prime tissues for fat use, pancreatic insulin secretion, release of gut satiety peptides, chylomicron secretion, and transport by the lymph.
5. Free fatty acid receptors 1 and 4 (FFAR1, FFAR4), like CD36, are activated by long-chain FAs and initiate signaling with pathways and outcomes similar to those elicited by FA/CD36. Understanding the potential interaction between CD36 and FFARs would be valuable.
6. CD36 signal transduction regulates the cellular fuel switch between glucose and FAs through influencing FA oxidation and insulin-stimulated glucose metabolism.
7. CD36-mediated increase of FA uptake and FA oxidation is important for integrity of the endothelial and epithelial barriers, which prevents pathogen and immune tissue infiltration and inflammation. CD36 deletion in lymphatic endothelial cells leads to lymph leakage, visceral obesity, and glucose intolerance.
8. Recent evidence supports the critical role of FA oxidation in progenitor cell upkeep and tissue renewal. Reduced FA uptake/oxidation impairs parietal cell progenitor differentiation and mucosal renewal after injury in stomachs of *Cd36*^−*/*−^ mice.
